# Tocotrienol in the Management of Nonalcoholic Fatty Liver Disease: A Systematic Review

**DOI:** 10.3390/nu15040834

**Published:** 2023-02-06

**Authors:** Kok-Yong Chin, Sophia Ogechi Ekeuku, Deborah Chia Hsin Chew, Anne Trias

**Affiliations:** 1 Department of Pharmacology, Faculty of Medicine, Universiti Kebangsaan Malaysia, Kuala Lumpur 56000, Malaysia; 2 Department of Medicine, Faculty of Medicine, Universiti Kebangsaan Malaysia, Kuala Lumpur 56000, Malaysia; 3 American River Nutrition, Hadley, MA 01035, USA

**Keywords:** hepatitis, metabolic disorders, steatosis, steatohepatitis, vitamin E

## Abstract

The increasing burden of nonalcoholic fatty liver disease (NAFLD) requires innovative management strategies, but an effective pharmacological agent has yet to be found. Apart from weight loss and lifestyle adjustments, one isomer of the vitamin E family—alpha-tocopherol—is currently recommended for nondiabetic steatohepatitis patients. Another member of the vitamin E family, tocotrienol (T3), has anti-inflammatory and antioxidant properties that reach beyond those of alpha-tocopherol, making it a potential agent for use in NAFLD management. This systematic review aimed to provide an overview of the effects of T3 supplementation on NAFLD from both clinical and preclinical perspectives. A literature search was performed in October 2022 using PubMed, Scopus and Web of Science. Original research articles reporting NAFLD outcomes were included in this review. The search located 12 articles (8 animal studies and 4 human studies). The literature reports state that T3 isomers or natural mixtures (derived from palm or annatto) improved NAFLD outcomes (liver histology, ultrasound or liver profile). However, the improvement depended on the severity of NAFLD, study period and type of intervention (isomers/mixture of different compositions). Mechanistically, T3 improved lipid metabolism and prevented liver steatosis, and reduced mitochondrial and endoplasmic reticulum stress, inflammation and ultimately liver fibrosis. In summary, T3 could be a potential agent for use in managing NAFLD, pending more comprehensive preclinical and human studies.

## 1. Introduction

Nonalcoholic fatty liver disease (NAFLD) describes a spectrum of liver diseases ranging from benign fatty liver to advanced nonalcoholic steatohepatitis (NASH) with the exclusion of alcohol consumption as an aetiology [1]. If left untreated, NASH will progress to cirrhosis and end-stage liver disease requiring liver transplantation, or to hepatocellular carcinoma. It is also associated with an increased risk for cardiovascular diseases (CVDs), type 2 diabetes, chronic renal disease and extrahepatic cancers [2,3]. The gold standard for NAFLD diagnosis is liver biopsy, but the advent of biochemical markers (e.g., alanine aminotransferase), imaging techniques (e.g., ultrasound, computed tomography and magnetic resonance imaging), combinatorial predictive algorithms (e.g., Fibrosis-4 index (FIB4) and NASH diagnostic panel) and transient elastography (FibroScan^®^, which measures both liver steatosis and liver fibrosis concurrently) has allowed noninvasive identification of the condition [4].

Metabolic syndrome, characterised by a concurrent presence of central obesity (waist circumference > 40 inches for men and > 35 inches for women), hypertension (blood pressure > 130/85 mmHg), hyperglycaemia (fasting blood glucose > 100 mg/dL) and dyslipidaemia (fasting triglycerides (TG) > 150 mg/dL, and fasting high-density lipoprotein cholesterol < 40 mg/dL in men and < 50 mg/dL in women) [5,6], is a strong risk factor for NAFLD [7]. The morbidity and mortality of NAFLD’s extrahepatic complications could be attributed to risk factors shared with metabolic syndrome because of its association with CVDs and type 2 diabetes. In a meta-analysis, incident metabolic syndrome was reported to coincide with NAFLD defined by ultrasonography [3.22 (95% CI, 3.05–3.41)] [8]. The global prevalence of NAFLD was estimated at 32.4% (95% CI 29.9–34.9), with male predominance and an increasing trend over time [9]. This is not surprising as the prevalence of metabolic syndrome has also been increasing over the years due to the rapid westernisation of lifestyle and diet in developing countries [10].

The classic theory explaining the pathogenesis of NAFLD is based on the two-hit hypothesis, wherein the first hit is lipid accumulation due to insulin resistance, which increases the susceptibility of the liver to injury. The second hit stimulates hepatic injury, inflammation and fibrosis [11,12]. However, this simplistic hypothesis is outdated and has been replaced with the multiple-hit hypothesis, which indicates that various factors act concurrently or sequentially in the development of NAFLD. A combination of dietary (e.g., a high-fat and/or high-fructose diet) and lifestyle (e.g., lack of physical activity) factors can increase circulating free fatty acid and cholesterol levels, leading to insulin resistance, expansion of adipose tissue and an altered gut microbiome. The release of pro-inflammatory cytokines and adipokines, as well as lipolysis from adipose tissue, further aggravate insulin resistance, which stimulates de novo fatty acid synthesis by the liver. Consequently, the influx of free fatty acid causes TG accumulation and lipotoxicity of the liver, leading to mitochondrial dysfunction and the generation of free radicals. Concurrently, endoplasmic reticulum stress is induced, leading to the unfolded protein response. Both processes lead to hepatic inflammation and subsequent fibrosis [13,14,15,16].

There is an unmet need for NAFLD pharmacotherapy. Various trials investigating drugs targeting insulin signalling (e.g., metformin), bile acid signalling (e.g., obeticholic acid) and lipid metabolism (e.g., peroxisome proliferator-activator receptor α/δ agonists) in the liver are currently ongoing [17,18]. Vitamin E has received much attention as a potential treatment agent for NAFLD due to its biological properties as an anti-inflammatory and antioxidant agent. Multiple meta-analysis reports have also indicated that vitamin E can improve the metabolic profile, liver enzyme levels and liver pathology of patients with NAFLD [19,20,21,22]. Most of these studies used α-tocopherol (αTF) as the treatment agent, leaving another potent vitamin E family, tocotrienol (T3), unexplored.

Vitamin E can be categorised into two families, i.e., TF and T3. Each of them contains four isomers (α-, β-, γ- and δ-), differentiated based on the position of the methyl groups on the chromanol ring. T3 can be found abundantly in botanical oils from palm, rice bran and annatto bean [23]. In natural sources, T3 and αTF coexist together in varying compositions [24]. For example, the annatto vitamin E mixture consists of mainly T3 with a negligible amount of αTF [25]. Palm T3-rich fraction consists of 20–30 % αTF and the rest is T3 [26]. Despite sharing the common chromanol ring, T3 differs from TF due to the unsaturated isoprenoid side chain [27]. This characteristic gives T3 some unique properties, such as better antioxidant effects on a lipid bilayer, and mevalonate-suppressive/cholesterol-lowering effects [28]. Previous studies have also established that T3 is a potent agent against metabolic syndrome and its constituent derangements [29,30,31]. Therefore, T3 could be a promising agent for use in suppressing NAFLD.

This systematic review aims to summarise the current knowledge on the effects of T3 against NAFLD to position T3 in the management of NAFLD to reduce the burden of this condition.

## 2. Methodology

This systematic review was conducted based on the Preferred Reporting Items for Systematic Reviews and Meta-Analyses (PRISMA) guidelines [32] (Table S1). The protocol of this systematic review was registered in the Open Science Framework Registries (access link: https://doi.org/10.17605/OSF.IO/PKYA9). The search string used in the literature search conducted in October 2022 was (tocotrienol) AND (non-alcoholic OR nonalcoholic) AND (“fatty liver disease” OR steatohepatitis OR steatosis). Electronic databases, i.e., PubMed, Scopus and Web of Science, were used in the literature search. All records from the inception of the databases were searched. Reference tracing of the included articles was conducted to confirm all relevant literature was included.

The current review included all original research articles exploring the therapeutic effects of T3 on NAFLD. The studies included patients clinically diagnosed with NAFLD or animal models of NAFLD. The interventions given consisted of pure T3 isomers or natural mixtures. The inclusion of patients or animals treated with placebo was necessary, but a positive control was not compulsory because there is no standard therapy for NAFLD. If a placebo group was absent, the pretreatment levels of outcomes of interest were required for a study to be considered. The included studies also reported NAFLD outcomes (liver profile, histology or imaging outcomes). Both preclinical and clinical studies were considered. Articles not written in English or not containing original data (reviews, letters, commentaries or opinions) were excluded. Studies using a mixture of T3 and other non-vitamin-E compounds were excluded because the effects of T3 could not be delineated. Conference abstracts and proceedings were excluded to avoid duplication with the original articles.

The organisation of literature and identification of duplicated items were performed using Endnote X9 (Clarivate, Philadelphia, USA). Screening of articles’ titles and abstracts was performed by K.-Y.C. and A.T. Full texts of articles passing this stage were retrieved and examined in detail, guided by the inclusion and exclusion criteria. The third author (S.O.E.) was consulted if any discrepancies arose. K.-Y.C. and S.O.E. performed data extraction using a standardised table. The information retrieved included authors, year, study design, major findings and limitations. D.C.H.C. validated the content of the current review.

## 3. Results

**Figure 1:**
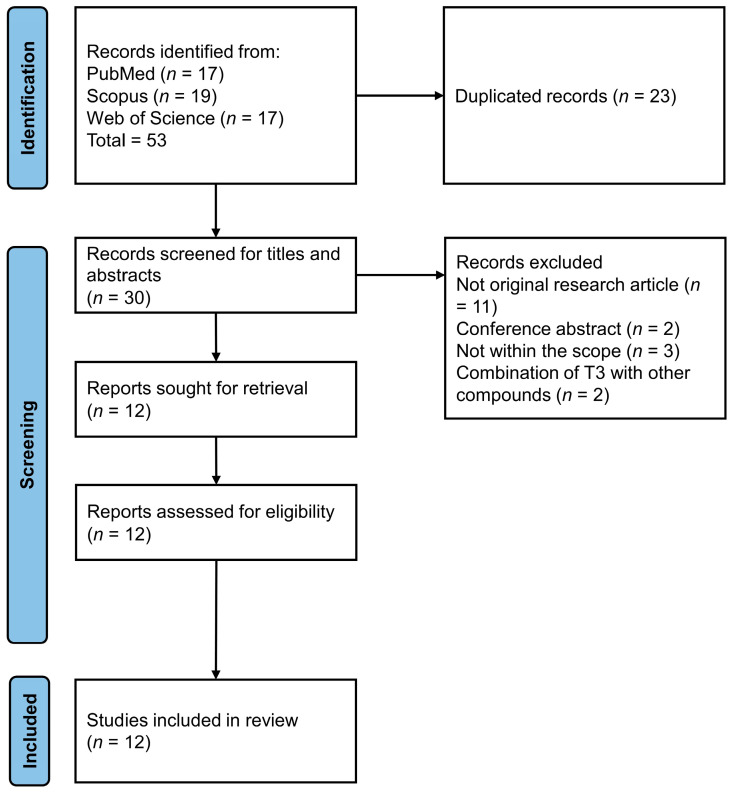
Flow chart of the article selection process.

The literature search revealed 53 articles (PubMed = 17, Scopus = 19 and Web of Science = 17), of which 23 were identified as duplicates and removed. Of the remaining 30 items, 11 did not contain primary data, 2 were conference abstracts, 3 were beyond the scope of the current review and 2 studies used mixtures of T3 with other compounds. Twelve articles were included for full-text screening and included in the current review. The article screening and selection process is depicted in Figure 1.

Of the 12 studies included, 8 were animal studies and 4 were human studies. The animal studies used normal rodents fed with a high-fat or high-fat high-carbohydrate diet for 14–20 weeks to induce metabolic syndrome and subsequent NAFLD [33,34,35]. Yachi et al. induced NAFLD in rats with a vitamin-E-deficit high-fat diet for 4 weeks, followed by administration of tumour necrosis factor-α (TNF-α) and D-galactosamine 2–5 h before sacrifice to mimic NAFLD changes [36]. This model was based on the classic two-hit hypothesis, wherein high-fat-diet-induced fat accumulation represented the first hit, and TNF-α/D-galactosamine-induced inflammation represented the second hit [36]. Two studies used a choline-deficient and L-amino-acid-deficient (CDAA; 3 weeks) or methionine and choline-deficient diet (MCD; 12 weeks) to induce NAFLD in mice [37,38]. MCD disturbed β-oxidation of fatty acid and VLDL secretion, leading to liver damage without insulin resistance [39]. This model helps to illustrate the development of NASH without manifested insulin resistance [37], although this condition is rare clinically [40]. The protein content of CDAA was replaced with an equivalent mixture of L-amino acid, and in combination with a high-fat diet as used by Noichi et al., induced NAFLD rapidly [38]. In the studies of Kim et al., a C/EBP homologous protein (CHOP)-knockout mouse model fed with the MCD diet was used to validate the role of T3 in suppressing endoplasmic reticulum stress [37]. Another genetically modified mouse model used was B6.Cg-LepOb/J mice, which develop metabolic syndrome and obesity spontaneously. They were fed a high-fat diet for 6 weeks to induce NAFLD [41]. The T3 treatments and their durations have been summarised in Table 1.

The four human clinical trials were conducted by two groups of researchers. Magosso et al. supplemented patients with NAFLD with 400 mg palm T3 daily for 1 year and assessed the hepatic echogenic response [42]. Pervez et al. supplemented patients with NAFLD with 600 mg annatto T3 daily for 12–48 weeks [43,44,45]. The patients were advised to consume a low-fat diet and exercise at the same time. The outcomes assessed included fatty liver index, serum inflammation markers, antioxidant markers and miRNA expression. The latest study by Pervez et al. compared the efficacy of 600 mg annatto T3 and 800 IU αTF for 48 weeks [44]. This study did not have a placebo control group, but the pre-treatment outcomes for each treatment arm were included.

In summary, animal studies revealed that T3 isomers and natural mixtures improved the liver profile and attenuated TG accumulation in the liver. They also improved NAFLD activity scores and liver histology. Wong et el. found that the δT3 isomer was the most potent in suppressing metabolic derangements in rats fed with HCHF diets, but all vitamin E isomers studied (αTF, αT3, γT3 and δT3) reduced lipid droplets and inflammatory cell infiltration and improved liver profile [46]. Kim et al. found that γT3 reduced hepatic fibrosis with minimal impact on steatosis in mice fed with the MCD diet, despite the downregulation of de novo lipogenesis, inflammation and fibrosis markers [37]. They further confirmed that the protective action of γT3 was mediated by the reduction of endoplasmic reticulum stress using CHOP-knockout mice [37]. Annatto T3 was reported to increase fatty acid oxidation while reducing fatty acid synthesis in the liver and adipose tissues [33]. Both annatto T3 and palm T3 were reported to suppress Toll-like receptor signalling and nuclear factor kappa-B signalling and reduce oxidative stress [35]. A metabolomic study in B6.Cg-LepOb/J mice fed with a high-fat diet revealed that palm T3 decreased arachidonic acid and sphingosine-1-phosphate (responsible for inflammation), N-undecanoylglycine and ketogenic amino acid (markers of impaired fatty acid oxidation).

In human studies, palm T3 normalised the hepatic echogenic response and prevented the worsening of steatotic grade in the supplemented patients [42]. Annatto T3 improved liver profile, fatty liver index, circulating inflammation and lipid peroxidation markers in patients starting from 12 weeks of supplementation [43,45]. Although the 12-week treatment did not improve hepatic steatosis as assessed using ultrasound imaging [43], 24-week treatment with annatto T3 did, evident through a one-degree reduction in 10 patients and a two-degree reduction in one patient, compared to only four patients improving by one degree in the placebo group [45]. Twenty-four weeks of annatto T3 treatment further downregulated miR-122-5p, miR-34a-5p and miR-375-3p, responsible for lipid metabolism, and decreased insulin resistance, oxidative stress and inflammation [45]. In a 48-week study comparing αTF with annatto T3 supplementation in NAFLD, both treatments improved fatty liver index and glycaemic status, as well as inflammatory and oxidative stress markers [44], but annatto T3 was significantly more effective at decreasing body weight, circulating inflammatory cytokines and cytokeratin-18 fragment M30 (a hepatocyte apoptosis marker) than αTF [44].

The summary of the results is presented in Table 1.

**Table 1:** Summary of the effects of T3 on NAFLD.

Authors	Study Design/Model	Treatment	Major Findings
Preclinical studies			
Yachi et al. (2013) [36]	Animal: Male SD-IGS rats (7 weeks old) Disease model: TNF-α/D-galactosamine-induced steatohepatitis (induction 2 or 5 h before sacrifice)	αTF (0.85% food mass), T3 (0.8% food mass) or mixed αTF + T3 (0.85% +0.8% food mass)-enriched high-fat diet for 4 weeks (oral)	↓ liver TG content at 2 and 5 h in αTF + T3 group; ↔ in αTF and T3 alone group vs. negative control. ↑ liver MTP mRNA expression in αTF; ↔ liver MTP mRNA expression in T3 or αTF + T3 vs. negative control. ↓ plasma liver damage markers (AST, ALT) and TG in all treatment groups vs. negative control after 5 h. ↓ TBARS at 2 and 5 h in αTF + T3 group; ↔ in αTF or T3 vs. negative control. ↓ IL-1β at 2h in αTF and αTF + T3 group vs. negative control; ↔ IL-1β at 5h in all treated groups vs. negative control ↓ IL-6 at 2 h in all treated groups; at 5h in T3 and αTF + T3 groups vs. negative control ↓ liver TGF-β1 in αTF + T3 group vs. negative control at 2 h
	Cells: Primary hepatocytes from male SD-IGS rats (3 weeks old) Treatment: Induction: TNFα (0.1, 0.5 nM) for 2–12 h before hepatocyte harvest.	Hepatocytes from vitamin-E-supplemented rats (doses as indicated above)	↓ IL-1β and IL-6 mRNA expression in both αTF and αT3 after 8 h vs. negative control ↓ decline of MTP in αT3 vs. αTF and negative control. ↔ SMAD3/7/TGF-β mRNA expression in treatment vs. negative control.
Allen et al. (2017) [33]	Animals: Male C57BL/6J mice (age: 6 weeks old) Disease model: High-fat-diet-induced steatohepatitis	400 or 1600 mg/kg diet δT3 (or 28 or 112 mg/kg bw) for 14 weeks (oral)	↑ glucose tolerance; ↓ hepatic steatosis (TG droplets and macrophage infiltration), serum TG, fat cell size and macrophage infiltration vs. negative control ↔ BW, fat pad weight vs. negative control ↓ mRNA and protein expression of pro-inflammatory adipokines (TNF-α, IL-6, MCP-1 & leptin); ↑ expression of anti-inflammatory adipokines (IL-10) for 400 mg/kg δT3 vs. negative control. ↑ markers of fatty acid oxidation (mRNA level of CPT1A/2 (1600 mg/kg only), Forkhead box A2) vs. negative control in adipose tissue ↑ markers of fatty acid oxidation (mRNA level of PPARα, PPAR δ (400 mg/kg only) and CPT2 (400 mg/kg only)); ↔ in CTP1A vs. negative control in the liver tissue. ↓ markers of fatty acid synthesis (mRNA level of fatty acid synthase and acetyl-CoA carboxylase-1) in adipose tissue vs. negative control ↓ markers of fatty acid synthesis (FASN, SCD-1 (400 mg/kg only), pyruvate kinase (1600 mg/kg only) and carbohydrate-responsive element-binding (1600 mg/kg only)) in liver tissue vs. negative control
Wong et al. (2017) [46]	Animals: Male Wistar rats (age: 9–10 weeks old) Disease model: HC- or HCHF-diet-induced metabolic syndrome	85 mg/kg bw of αTF, αT3, γT3, δT3 for 8 weeks (oral)	↓ total fat mass, abdominal circumference, adiposity index and retroperitoneal and epididymal fat pads vs. HCHF control in δT3 ↓ total cholesterol, NEFA, TG in δT3 vs. HCHF control, αTF, αT3 and γT3; ↓ plasma NEFA in αTF, αT3, γT3 vs. HCHF control. ↓ lipid droplets, portal inflammatory cell infiltration and ALT, AST level in all treated groups ↓ fasting plasma glucose levels, postprandial blood and glucose AUC in OGTT test in δT3
Kim et al. (2018) [37]	Animals: Male C57BL/6J mice (age: 6 weeks old) Disease model: High fat (45%), cholesterol (0.2%) and sucrose (in drinking water) (HFCS)-diet-induced NAFLD/NASH	γT3 (0.1% food mass) for 12 weeks (oral)	↓ body weight; ↑ daytime activity, energy expenditure and respiratory exchange ratio vs. negative control ↓ total cholesterol, LDL, fasting glucose, fasting insulin, plasma lactate and higher GSH/GSSG ratio and glucose clearance vs. negative control ↓ TG content and lipid droplets in liver vs. negative control ↓ pro-inflammatory cytokines mRNA expression (MCP-1, CD11c, TNF-α, NLRP3 and IL-1β) in liver ↓ de novo lipogenic gene expression (PPARγ, SREBP1C, FAS, DGAT2, SCD1 and LPL, DNL, ACC), ER stress protein expression (BiP, CHOP, p-JNK, peIF2α and p-p38) and fibrosis-related gene expression (α-SMA, TIMP-1, TGF- α, COL1a1 and HDAC9) in the liver vs. negative control ↓ mRNA expression (MCP1, IL-1β, IL-18 and IL-6) and protein expression (IκBα, p-p38 and p38) of inflammatory markers in the pancreas vs. negative control. ↓ mRNA expression (F4/80, MCP-1, TNF-α, IL-1β and IL-18) and protein expression (F4/80) of pro-inflammatory markers in the epididymal fat.
	Animals: Male C57BL/6J mice (age: 6 weeks old) Disease model: methionine- and choline-deficient diet (MCD)-induced NAFLD/NASH	γT3 (0.1% food mass) for 5 weeks (oral)	↔ total hepatic TG content; ↓ fibrosis vs. negative control ↓ hepatic stress and fibrosis with minimal impact on steatosis vs. negative control ↓ lipogenesis, ER stress, inflammation and fibrosis vs. negative control
	Animal: Male CHOP-knockout mice (age: 6 weeks old) Disease model: MCD-induced NAFLD/NASH	γT3 (0.1% food mass) for 5 weeks (oral)	↑ protective effects of γT3 due to CHOP deletion vs. negative control ↓ hepatic fibrosis and inflammation vs. negative control
Wong et al. (2020) [28]	Animals: Male Wistar rats (age: 12 weeks old) Disease model: HCHF-diet-induced NAFLD	60 or 100 mg/kg bw of AnT3 (with 16% γT3 and 84% δT3) or pT3 (21.9% αTF, 24.7% αT3, 4.5% βT3, 36.9% γT3 and 12.0% δT3) for 12 weeks (oral)	↓ liver index in 60 mg/kg pT3 ↓ liver TLR 2 and IL-10 expression and serum CRP level in all T3 treatments except 100 mg/kg pT3 vs. negative control ↔ TLR4 expression vs. negative control ↓ total NFκB in all T3 treatments except 60 mg/kg pT3 vs. negative control ↑ Cytoplasmic p-NFκB/total NFκB in the AnT3 groups vs. negative control ↓ MDA level and ↑ SOD activity in all T3 groups; ↑ GPx activity in AnT3 and 60 mg/kg pT3; ↑ GSH content in 60 mg/kg pT3 vs. negative control ↓ steatosis score in 100 mg/kg pT3 100 vs. negative control ↔ % of red oil stain with treatment vs. negative control
Goon et al. (2021) [41]	Animals: Male B6.Cg-LepOb/J mice (age: 8 weeks old) Disease model: NAFLD induced using genetically modified mice through HFD	200 mg/kg TRF bw (72% T3 and 28% TF) or eTRF with palm kernel oil as the carrier (81% T3 and 19% TF) for 6 weeks (oral)	↓ Bile acids, lysine, arachidonic acid and sphingolipids; ↑ xanthine and hypoxanthine vs. negative control ↑ FXR IHC score vs. carrier and negative control ↑ FXR expression in eTRF vs. negative control ↓ NAFLD activity score vs. negative control
Goon et al. (2022) [34]	Animals: ICR mice (age: 6 weeks old) Disease model: HFD-induced NAFLD	200 mg/kg bw TRF (80% T3 and 20% TF) for 10 weeks (oral)	↔ body weight, waist circumference, random blood glucose vs. negative control↓ NAFLD activity score vs. negative control ↓ steatosis of parenchymal, hepatocyte ballooning and lobular inflammation vs. negative control ↓ fatty liver macroscopically and microscopically vs. negative control
Noichi et al. (2022) [38]	Animals: Male C57BL/6J mice (age: 5 weeks old) Disease model: CDAHFD-induced NAFLD/NASH	αTF (4 mg fixed dose) and T3 (32% αT3, 5% βT3, 48% γT3 and 15% δT3; 4 mg fixed dose) for 2 weeks (oral)	↔ liver weight, liver/body weight ratio, adipose tissue weight (epididymal, perirenal, mesenteric) vs. negative control ↑ TG in T3 group vs. negative control; ↑ ALT level in αTF vs. T3 and negative control; ↑AST level αTF vs. T3. ↓ F4/80, TNFα and Col4a1 in T3. ↔ TNF-α, Col1a1, α-SMA vs. negative control. ↓ MMP3 but not MMP13 in T3. ↓ dense collagen fibres vs. negative control
Human randomised controlled trials			
Magosso et al. (2013) [42]	Participants: Adults of both genders (age: 35 yrs and above) Disease: mild untreated hypercholesterolaemia and ultrasound-proven NAFLD	Treatment: 200 mg mixed tocotrienol (61.5 mg αT3, 112.8 mg γT3, 25.7 mg δT3, 61.1 mg αTF) twice daily for 1 year (oral). Placebo control: content not disclosed.	Normalisation of hepatic echogenic response vs. placebo Worsening of steatotic grade in 2 cases in the placebo group, none in the T3 group. ↑ decline of ApoB vs. placebo ↑ circulating T3 level vs. placebo
Pervez et al. (2018) [43]	Participants: Adults of both genders (age: > 20 yrs) Disease: ultrasound-proven fatty liver disease	Treatment: 300 mg AnT3 (90% δT3 and 10% γT3) twice daily for 12 weeks (oral). Placebo control: sucrose	↓ serum AST, hs-CRP, MDA and FLI score vs. placebo ↔ hepatic steatosis (ultrasound) vs. placebo. No adverse effect detected.
Pervez et al. (2020) [45]	Participants: Adults of both genders (age: > 20 yrs) Disease: ultrasound-proven fatty liver disease	Treatment: 300 mg AnT3 (90% δT3 and 10% γT3) twice daily for 24 weeks (oral). Placebo control: sucrose	↓ FLI, HOMA-IR, hs-CRP, IL-6, TNF-α, MDA, AST, ALT. ↓ expression of miR-122-5p, miR-34a-5p and miR-375-3p No adverse events reported.
Pervez et al. (2022) [44]	Participants: Adults of both genders (age: 20–70 yrs) Disease: NAFLD	Treatment: 300 mg δT3 or 268 mg αTF twice daily for 24 and 48 weeks (oral). Placebo control: no	Improved FLI, liver-to-spleen CT attenuation ratio, HOMA-IR, serum hs-CRP and malondialdehyde ratio for both groups compared to baseline, but not significant between them. ↓ body weight, IL6, TNFα, leptin, cytokeratin-18 fragment M30; ↑ adiponectin in δT3 vs. αTF at 48 weeks

Abbreviation: ↔, no change; ↑, increase; ↓, decrease; AnT3, annatto tocotrienol; pT3, palm tocotrienol; αTF, alpha tocopherol; αT3, alpha tocotrienol; βT3, beta tocotrienol; γT3, gamma tocotrienol; δT, delta tocotrienol; bw, body weight; TNF-α, tumour necrosis factor alpha; TG, triglyceride; NAFLD/NASH, nonalcoholic fatty liver disease/ nonalcoholic steatohepatitis; CPT1A/2, carnitine palmitoyltransferase1A/2; HC, high-carbohydrate; HCHF, high-carbohydrate high-fat; CHOP/, B6.129 (Cg)-Ddit3tm2.1Dron/J, homozygous knockout for C/EBP homologous protein in the C57BL/6 background LF, low-fat; HFCS, high-fat, -cholesterol and -sugar; FXR, Farnesoid-X Receptor; CDAHFD, choline-deficient L-amino-acid-deficient high-fat diet; IL-1β/6/8/10, interleukin 1 beta/6/8/10; TGF-β1, transforming growth factor-β 1; TBARS, thiobarbituric-acid-reactive substances; ALT, alanine transferase; AST, asparagine transferase; MTP, microsomal triglyceride transfer protein; MCD, methionine- and choline-deficient diet, MDA, malondialdehyde, hs-CRP, high-sensitivity C-reactive protein; FLI, fatty liver inhibition; HOMA-IR, Homeostatic Model Assessment for Insulin Resistance, ApoB, apolipoprotein B; VLDL, very-low-density lipoprotein; AUC, area under curve; OGTT, oral glucose tolerance test; IHC, immunohistochemistry; eTRF, enhanced tocotrienol-rich fraction; GSH, glutathione reductase; GPx, glutathione peroxidase; SOD, superoxide dismutase; p-NFkB, phosphorylated nuclear factor kappa-light chain enhancer of activated B cells Col1a1/4a1, collagen type 1 a1/type 4 a1; α-SMA, alpha-smooth muscle actin; MCP-1, monocyte chemotactic factor-1; PPARα/δ/γ; peroxisome proliferator-activated receptor alpha/delta/gamma; FASN, Fatty acid synthase; SCD-1, stearoyl-CoA desaturase 1; NEFA, non-esterified fatty acid; SREBP1C, Sterol regulatory element-binding protein-1c; oxidised glutathione (GSSG); FAS, fatty acid synthase; DGAT2, Diacylglycerol-O-acyltransferase 2; LPL, lipoprotein lipase; DNL, de novo lipogenesis; ACC, acetyl-CoA carboxylase; BiP, binding-immunoglobulin protein; CHOP, CCAAT-enhancer-binding protein homologous protein; p-JNK, phosphorylated c-Jun amino-terminal kinases; peIF2α, phosphorylated, eukaryotic initiation factor-2 alpha; Timp-1, Tissue inhibitor of metalloproteinases-1; Hdac9, Histone deacetylase 9; IκBα, nuclear factor of kappa light polypeptide gene enhancer in B-cells inhibitor, alpha; F4/80; ER, endoplasmic reticulum; TLR2/4, toll-like receptor 2/4; MMP-3/13, matrix metalloproteinase-3/13.

## 4. Discussion

Despite the outdated two-hit hypothesis, the progression of NAFLD from steatosis to steatohepatitis and liver fibrosis has been replicated in animal models [47]. TG accumulation in the liver, visible as lipid droplets in the cytoplasm of hepatocytes, is the initial manifestation of NAFLD. TG accumulation occurs due to insulin resistance, which promotes lipolysis of adipose tissue and the influx of nonesterified fatty acids (NEFAs) into the liver. De novo lipogenesis of the liver, promoted by hyperinsulinemia and excessive fructose intake, is also a contributor to TG accumulation in the liver [40]. Specifically, fatty acid synthase (FAS), acetyl-CoA carboxylase (ACC), stearoyl-CoA desaturase 1 (SCD1) and diglyceride acyltransferase 2 (DGAT2) control hepatic fatty acid synthesis and are under the influence of sterol regulatory element-binding protein 1c (SREBP1c) (activated by insulin) and carbohydrate-responsive element-binding protein (ChREBP) (activated by glucose) transcriptionally [48]. PPARγ regulates the expression of lipogenic enzymes indirectly through SREBP1c [49]. PPARγ also directly regulates the expression of lipoprotein lipase (LPL), which hydrolyses triacylglycerol-rich lipoproteins (TRLs) to release fatty acids for cellular uptake [49]. The current evidence indicates that T3 may prevent TG accumulation in the liver, especially in NAFLD models induced by the HCHF diet [31,33,37]. γT3 has been reported to reduce the expression of PPARγ, SREBP1c, ACC, FAS, DGAT2, SCD1 and LPL involved in de novo lipogenesis [37]. Palm T3 also triggered a similar response (reduced expression of ChREBP, ACC, FAS, SCD1 and pyruvate kinase) in the adipose tissues and livers of mice fed with a high-fat diet [33].

Previous studies suggest that the antisteatosis effects of T3 are dependent on the severity of the disease and the treatment period. Kim et al. illustrated in a parallel study that γT3 only reduced liver TG content and lipid droplets in the HCHF model, and not in the MCD model [37]. A contributing reason for this discrepancy is probably the severity and pathomechanism of NAFLD of the models used; insulin resistance was absent in the MCD model. Montandon et al. reported that a high-fat atherogenic diet stimulated counter-regulatory mechanisms in the liver lipid metabolism, while the MCD diet aggravated TG accumulation by increasing lipid import and lowering lipid export concurrently [50]. The MCD diet also induced a significant alternation in liver phospholipid composition compared to the atherogenic diet [50]. Moreover, although fatty liver biomarkers decreased significantly, Pervez et al. did not detect ultrasound-confirmed improvement in hepatic steatosis among patients with NAFLD after supplementation of annatto T3 (600 mg daily) for 12 weeks [43], whereas hepatic steatosis improvement was noted after 24 weeks at the same dose [45]. This observation implies that a longer supplementation period is necessary.

Fatty acids in hepatocytes undergo β-oxidation in the mitochondria [51]. Fatty acids are converted to acyl-CoA by acetyl-CoA synthetase, then transported to the mitochondrial matrix by the carnitine shuttle, comprising carnitine-palmitoyltransferase 1 (CPT1), carnitine-acylcarnitine carrier (CAC) and carnitine-palmitoyltransferase 2 (CPT2). Acyl-CoA undergoes β-oxidation to produce acetyl-CoA, which is metabolised further in the tricarboxylic acid cycle [52]. Mitochondria may initially be able to cope with the influx of fatty acids by increasing oxidation and upregulating the mitochondrial respiratory chain. However, this process generates reactive oxygen species (ROS), which may overwhelm the cellular antioxidant mechanism, leading to oxidative stress. Oxidative stress can cause cellular damage and aggravate NAFLD. As the condition progresses, mitochondria dysfunction, marked by decreased ATP output and increased ROS generation, will occur [53,54]. Delta-T3 has been reported to improve fatty acid oxidation by upregulating mRNA levels of CTP1A, CTP2 and Forkhead box A2 (a positive regulator of genes involved in lipid oxidation) in the adipose tissue and liver in mice fed with a high-fat diet [33].

The endoplasmic reticulum (ER) is an organelle sensitive to the negative effects of oxidative stress. ER stress can result in the accumulation of unfolded and misfolded proteins, which trigger unfolded protein response. The failure of this protein can result in inflammation and further oxidative stress [55]. γT3 has been shown to improve ER stress in mice fed with the HCHF diet or MCD diet, marked by reduced binding immunoglobulin protein, CHOP, as well as phosphorylated c-Jun N-terminal kinases, eukaryotic translation initiation factor 2 subunit 1 and p38. In addition, CHOP-knockout mice showed attenuated responses to the beneficial effects of γT3 [37].

A HCHF diet is known to alter the gut microbiome, leading to increased gut permeability and activation of the innate immune system in response to the leakage of pathogen-associated molecular patterns (PAMPs), such as lipopolysaccharides (LPSs), peptidoglycans, viral or bacterial DNA or fungal beta-glucans, which can activate the liver immune (Kupffer cells, dendritic cells, natural killer (NK) and NK T cells) and nonimmune cells (hepatic stellate cells and endothelial cells) and induce an inflammation reaction [56]. Toll-like receptors on these cells recognise the structurally conserved PAMPs, subsequently triggering MyD88-dependent and -independent responses. The MyD88-dependent response involves the activation of the NFκB pathway and translocation of pro-inflammatory cytokines such as TNFα, IL-6 and IL-1β [57]. Oxidative stress can activate the NFκB pathway as well [58]. The MyD88-independent pathway involves the phosphorylation of interleukin regulatory factor 3 and type I interferon induction [59]. The combination of pro-inflammatory and pro-oxidative environments can trigger steatohepatitis via the activation of Kupffer cells and hepatic stellate cells. This steatohepatitis can progress to fibrosis and ultimately cirrhosis [60]. Wong et al. demonstrated that both annatto and palm T3 suppressed TLR3 but not TLR4 expression in the liver [35]. Both natural T3 mixtures also reduced total NFκB levels, but only annatto T3 increased cytoplasmic p-NFκB/total NFκB levels [35]. However, Wong et al. did not further characterise the gut microbiome. Several studies have shown that T3 can alter the gut microbiome in animal models. Annatto T3 was reported to decrease *Firmicutes* to *Bacteroidetes* ratio and lower the abundance of *Ruminococcus lactaris*, *Dorea longicatena* and *Lachnospiraceae* family members in faecal samples of rats fed a high-fat diet. It also increased the abundance of *Akkermansia muciniphila*. The altered gut microbiome was accompanied by improved glucose homeostasis, and reduced resistin, leptin and IL-6 levels in the white adipose tissue of supplemented rats [61]. In a murine model of colorectal cancer, supplementation of turmeric essential oil–curcumin–palm TRF decreased *Bacteroidaceae* but not *Lactobacillaeceae* and *Bifidobactericeae* [62]. These studies highlight the potential of T3 to modify the gut microbiome, thereby exerting suppressive effects on systemic inflammation. Further validation in an animal model or patients with NAFLD would be useful to illuminate the microbiome-enhancing properties of T3.

In addition to its indirect action on chronic inflammation, T3 is known to possess anti-inflammatory and antioxidant effects by itself. Many of the studies included in the current review reported downregulation of pro-inflammatory cytokines and lipid peroxidation products, as well as upregulation of the antioxidant defence system in NAFLD models treated with T3 [35,36,37,44,45]. Mechanistically, γT3 was shown to inhibit LPS-induced production of IL-6 without altering TNFα, IL-10 and cyclooxygenase-2 in murine RAW264.7 macrophages. This was achieved by the blocking of NFκB and CCAAT/enhancer-binding protein activation by γT3 [63]. Delta-T3 was shown to inhibit TNFα-induced IL-6 production in RAW264.7 macrophages by inhibiting the phosphorylation of transforming growth factor β-activated kinase 1 upstream of NFκB and increasing the expression of A20 and cylindromatosis, which are inhibitors of NFκB [64]. Palm T3 was reported to increase nuclear translocation of nuclear factor erythroid 2–related factor 2, which is a transcription factor regulating the expression of various antioxidant enzymes [65]. These findings point towards the use of T3 to suppress inflammatory and oxidative stress conditions, including NAFLD.

Bile acid is a signalling molecule that acts on hepatic and extrahepatic tissues to regulate carbohydrate and lipid metabolism [66]. FXR, a bile acid receptor, is highly expressed in the liver, small intestines and kidneys, and is responsible for glucose and lipid metabolism [67]. FXR was shown to suppress steatosis by inhibiting SREBP1c and induction on PPARα and its target, CPT1 [68]. Palm TRF (unformulated or enhanced) was shown to increase the protein and mRNA expression of FXR in the liver [41]. However, no significant changes in mRNA expression of FXR targets, i.e., small heterodimer partner and signal transducer and activator of transcription 3, were observed between treated and untreated B6.Cg-LepOb/J male fed with a high-fat diet [41]. The authors postulated that the fatty acids in the vehicle for T3 could attenuate FXR signalling despite the increased FXR expression [41].

Fibrosis is a result of the wound-healing response of the liver following injury. It is characterised by the deposition of high-density extracellular matrix (ECM) proteins which disrupt the normal liver architecture [69]. Hepatic stellate cells, upon activation, will change morphology, proliferate and produce tissue inhibitors of metalloproteinases (TIMPs) and ECM [70]. The transforming growth factor beta (TGFβ)/SMAD signalling pathway is crucial in mediating fibrogenesis [71]. γT3 has been reported to reduce markers of fibrosis (αSMA, TIMP1, TGFβ, histone deacetylase 9) in mice fed with the HCHF and MCD diets [37]. Using C57BL/6J mice fed with CDAA and a high-fat diet, Nochi et al. demonstrated that αTF and palm T3 reduced dense collagen fibres in the liver [38]. However, only palm T3 suppressed the expression of genes coding for TNFα and collagen 1α1, which are critical in fibrogenesis. Metalloproteinase (MMP) 3, a marker of fibrinolysis, was also reduced with palm T3 treatment [38]. This could have been due to a lower amount of ECM to be lysed in the treated group.

As evidenced from clinical trials [43,44,45], the protective action of T3 against NAFLD is the culmination of multiple mechanisms, such as anti-inflammation (reduced IL-6 and TNFα) and antioxidant (reduced MDA) effects and correction of metabolic derangements. It is of note that the Homeostatic Model Assessment of Insulin Resistance (HOMA IR), which reflects the extent of insulin resistance, was reduced by 15% from baseline in patients with NAFLD supplemented with annatto T3 after 48 weeks [45]. This observation has been supported by multiple animal studies and human clinical trials [72,73]. Furthermore, annatto T3 also increased adiponectin levels by 44% and reduced leptin levels by 18% from baseline in patients with NAFLD after 48 weeks [45]. Both adiponectin and leptin are hormones secreted by adipose tissue. Adiponectin regulates glucose levels and fatty acid metabolism, whereas leptin exerts pro-angiogenic and pro-inflammatory effects on the body [74,75]. The alternation of both hormones mediated by annatto T3 could lead to improved metabolic status and attenuate the effects of insulin resistance on the liver.

There have been two animal studies and one human study comparing the efficacies of T3 and αTF in NAFLD management [36,38,44]. All studies agreed that T3 was more effective at attenuating the pathological changes of NAFLD. It is of note that in the clinical trial by Pervez et al., annatto T3 induced significantly greater weight loss and TG reduction compared to αTF [44]. It also exerted greater effects in downregulating pro-inflammatory (IL-6 and TNFα) cytokines, as well as modulating adiponectin and leptin levels [44]. Most importantly, the hepatocyte apoptosis marker cytokeratin-18 fragment M30 was markedly reduced in the annatto T3 group compared to the αTF group [44]. Other indicators such as liver enzyme profile, lipid peroxidation products and hs-CRP levels were reduced from baseline, but no statistically significant difference was noted between αTF and annatto T3 [44]. Currently, αTF is recommended for the treatment of NAFLD [76]. If T3 is proven to be more effective than αTF, it could be a better option for the management of NAFLD and further reduce the health burden of the disease.

The better efficacy of T3 compared to αTF could be attributed to several factors. T3 is reported to be a better antioxidant than αTF because of higher recycling efficiency from chromanoxyl radicals, more uniform distribution on the lipid membrane and stronger disordering of the membrane, which allows better interaction of chromanols with lipid radicals [77]. T3 also possesses mevalonate-suppressive activities, which lower the generation of isoprenoids that serve as substrates for the production of small GTPases (e.g., ras homologous family member A (RhoA) and rat sarcoma protein (Ras)) [78]. Simvastatin, another mevalonate pathway suppressor, has been shown to prevent fibrosis and inflammation in NAFLD by suppressing RhoA- and Ras-mediated pathways without affecting steatosis [79]. However, the liver-protective effects of T3 through this pathway have not been investigated.

Regarding safety, all RCTs conducted summarised in this review reported no adverse effects among patients with NAFLD supplemented with T3 up to 600 mg for 48 weeks [42,43,44,45]. Annatto and palm T3 have been granted generally recognised as safe (GRAS) status by the US Food and Drug Administration [80,81]. Subacute (14 days of supplementation) and subchronic (42 days of supplementation) toxicological evaluation in mice revealed that palm T3 at 500 and 1000 mg increased bleeding and clotting time [82]. A 13-week oral toxicity study in rats established that the no-observed-adverse-effect level (NOAEL) of palm T3 was 0.19% diet (male rats: 120 mg/kg body weight/day; female rats: 130 mg/kg body weight/day). Significant changes in haematological profile and liver function were observed at doses beyond the NOAEL [83]. Since patients with NAFLD might suffer from higher CVD risk and are typically prescribed antithrombotic agents, the use of T3 should be cautioned due to increased haemorrhagic risk, though this effect has not been shown in human studies.

It should be noted that all human studies conducted on this topic to date have been single-centre randomised controlled trials. All studies indicated blinding of patients and physicians, as well as the fate of participants (completed/withdrawn from the trials), but methods of randomisation were not consistently reported. The NAFLD status of the subjects was performed via ultrasound technique rather than MRI in all trials. Only one clinical trial used the hepatic echogenic response in evaluating the progression of NALFD [42], while the others relies on surrogate markers. These areas could be improved in future trials.

The review is not without its limitations. It did not include studies that investigated the antimetabolic effects of T3 (anti-obesity, antidiabetic, antihyperlipidaemic, antihypertensive and antiatherogenic) without a liver investigation [72,84,85]. These antimetabolic effects of T3 could help to ameliorate the risk of NAFLD. Although the review focused on the effects of T3 on NAFLD, it should be acknowledged that the palm T3 mixture contains a significant amount of αTF. It is challenging to delineate between the effects T3 and αTF in the mixture. Grey literature was not included as part of the search strategy, so potential studies published as theses, monographs or other forms were excluded. A meta-analysis was not performed due to the heterogeneity of the parameters assessed in each study.

## 5. Conclusions

**Figure 2:**
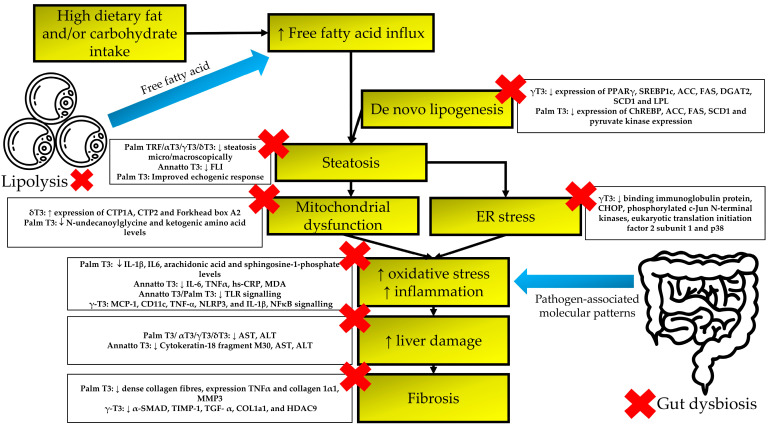
Mechanisms of T3-associated benefits in managing NAFLD. T3 has been shown to suppress lipolysis in adipose tissue, and prevent de novo lipogenesis, mitochondrial dysfunction and ER stress in the liver. These processes ameliorate steatosis and prevent further liver damage and fibrosis. The red crosses represent the action of T3.

T3 is a potential agent for use in NAFLD management. The current literature demonstrated that T3, either as single isomers or natural mixtures, protects against the steatosis, inflammation, oxidative stress and fibrosis that occur during the development of NAFLD (Figure 2). However, the protection is dependent on the severity of the condition, the treatment period and the type of intervention used (isomer/composition of natural mixture). Given its protective effects on metabolic derangements, as presented in multiple clinical trials, T3 could prevent the development of NAFLD as well. More mechanistic studies and RCTs would help to validate the role of T3 in NAFLD management.

## Data Availability

Not applicable.

## Supplementary Materials

The following supporting information can be downloaded at: https://www.mdpi.com/article/10.3390/nu15040834/s1, Table S1: PRISMA checklist [32].

## Abbreviation

ACC, acetyl-CoA carboxylase; ALT, alanine transaminase; AST, aspartate transaminase; CHOP, C/EBP homologous protein; COL1a1, collagen type 1 alpha 1; CTP1A/2, carnitine palmitoyltransferase1A/2; DGAT2, diglyceride acyltransferase 2; FAS, fatty acid synthase; HDAC9, histone deacetylase 9; hs-CRP, high-sensitivity C-reactive protein; IL-1β, interleukin-1β; LPL, lipoprotein lipase; MCP-1, monocyte chemoattractant protein-1; NF-κB, nuclear factor kappa B; NLRP3, NLR family pyrin domain containing 3; PPARγ, peroxisome proliferator-activated receptor-gamma; SCD1, stearoyl-CoA desaturase 1; SREBP1c, sterol regulatory element-binding protein-1c; T3, tocotrienol; TGF-β, transforming growth factor beta; TIMP-1, tissue inhibitor matrix metalloproteinase 1; TLR, Toll-like receptor; α-SMA, α-smooth muscle actin.
